# Genetic Incorporation of Human Metallothionein into the Adenovirus Protein IX for Non-Invasive SPECT Imaging

**DOI:** 10.1371/journal.pone.0016792

**Published:** 2011-02-09

**Authors:** J. Michael Mathis, Shilpa Bhatia, Alok Khandelwal, Imre Kovesdi, Stephen J. Lokitz, Yoshi Odaka, Amol M. Takalkar, Tracee Terry, David T. Curiel

**Affiliations:** 1 Gene Therapy Program, Departments of Cellular Biology and Anatomy, LSU Health Sciences Center, Shreveport, Louisiana, United States of America; 2 Biomedical Research Foundation of Northwest Louisiana, Shreveport, Louisiana, United States of America; 3 VectorLogics, Inc., Birmingham, Alabama, United States of America; 4 Department of Radiation Oncology, Washington University School of Medicine, St. Louis, Missouri, United States of America; The University of Chicago, United States of America

## Abstract

As the limits of existing treatments for cancer are recognized, clearly novel therapies must be considered for successful treatment; cancer therapy using adenovirus vectors is a promising strategy. However tracking the biodistribution of adenovirus vectors *in vivo* is limited to invasive procedures such as biopsies, which are error prone, non-quantitative, and do not give a full representation of the pharmacokinetics involved. Current non-invasive imaging strategies using reporter gene expression have been applied to analyze adenoviral vectors. The major drawback to approaches that tag viruses with reporter genes is that these systems require initial viral infection and subsequent cellular expression of a reporter gene to allow non-invasive imaging. As an alternative to conventional vector detection techniques, we developed a specific genetic labeling system whereby an adenoviral vector incorporates a fusion between capsid protein IX and human metallothionein. Our study herein clearly demonstrates our ability to rescue viable adenoviral particles that display functional metallothionein (MT) as a component of their capsid surface. We demonstrate the feasibility of ^99m^Tc binding *in vitro* to the pIX-MT fusion on the capsid of adenovirus virions using a simple transchelation reaction. SPECT imaging of a mouse after administration of a ^99m^Tc-radiolabeled virus showed clear localization of radioactivity to the liver. This result strongly supports imaging using pIX-MT, visualizing the normal biodistribution of Ad primarily to the liver upon injection into mice. The ability we have developed to view real-time biodistribution in their physiological milieu represents a significant tool to study adenovirus biology *in vivo*.

## Introduction

### Barrier to gene therapy approaches for cancer

As the limits of existing treatments for cancer are recognized, clearly novel therapies must be considered for successful treatment; cancer therapy using adenovirus (Ad) vectors is a promising strategy. The existing approaches to gene therapy/virotherapy of cancer can be divided into five broad categories: a) mutation compensation, b) molecular chemotherapy, c) genetic immunopotentiation, d) genetic modulation of resistance/sensitivity, and e) oncolytic therapy or virotherapy. Through 2009, Ad vectors were employed in a quarter of gene therapy clinical trials worldwide [1]. However tracking the biodistribution of Ad vectors *in vivo* is limited to invasive procedures such as biopsies, which are error prone, non-quantitative, and do not give a full representation of the pharmacokinetics involved.

### Current detection methods are inadequate for Ad vector systems

Several imaging studies have attempted to address this problem, including the use of positron emission tomography (PET) scanning to detect herpes simplex virus thymidine kinase (HSV-TK) as a reporter of oncolytic herpes simplex virus replication [2]. However, detection was restricted to infected cells expressing the reporter gene, which does not represent the physical distribution of the virus itself. Another group employed soluble hCEA and βhCG peptide markers as a way to monitor oncolytic measles virus therapy in mice, which correlated with therapeutic outcome but could not show viral localization [3]. Other conventional imaging systems for adenovirus based gene therapy have been designed to detect transgene expression of reporters such as green fluorescent protein (GFP) [4], somatostatin receptor type 2 (SSTR-2) [5], [6], sodium iodide symporter [7], luciferase [8], and HSV-TK [9]. Despite their utility for assessing gene delivery and expression, these reporters by themselves are not suitable for monitoring physical biodistribution. The major drawback to approaches that tag viruses with reporter genes is that these systems require initial viral infection and subsequent cellular expression of a reporter gene to allow non-invasive imaging.

### Capsid protein IX is a potential location for labeling Ads

To overcome this limitation, we devised a novel approach to incorporate the human metallothionein (MT) protein as a fusion to the Ad minor capsid protein pIX (pIX). This would allow us to directly label Ads and determine if this approach can be used to monitor virus delivery *in vivo* by non-invasive imaging. Recent work by several groups has defined the C-terminus of pIX as a locus presenting incorporated ligands on the virus surface. Protein IX is a small polypeptide of 140 residues (14.7 kDa) that acts as a cement protein to stabilize hexon-hexon interaction and therefore the capsid structure itself [10]. Four trimers of pIX interact with a group of nine (GON) hexons in each facet of the icosahedron [11], resulting in 240 copies of the protein per virion [12]. In addition, pIX has been implicated as a transcriptional activator of several viral and cellular TATA-containing promoters, including adenoviral E1A, E4, and major late promoters [13]. Based on the understanding the pIX C-terminus is surface exposed [14], [15], pIX has been exploited as a location to incorporate heterologous peptides (namely lysine octapeptide and polylysine) into its C-terminus for retargeting purposes [16]. A single chain variable fragment (scFv) against beta-galactosidase [17] and a single-chain T-cell receptor (scTCR) directed against the melanoma-associated antigen (MAGE) [18] were also successfully fused to pIX and assembled into virions. Based on these data, we also demonstrated that pIX would be a suitable location for incorporating reporter genes, such as firefly luciferase [19] and GFP [20], [21]. However, these fusions require optical imaging techniques that are not clinically compatible and may be limited by depth of light penetration.

### Non-invasive imaging using a pIX-MT fusion

MT is a ubiquitous, low molecular weight, metal-binding protein that participates in heavy metal metabolism and detoxification. Mammalian forms of MT bind seven metal ions in tetrahedral metal-thiolate clusters, including a commonly used medical isotope of technetium (^99m^Tc) useful for radioimaging by single photon emission computed tomography (SPECT) [22]. Several studies have shown that MT can be genetically engineered or conjugated with targeting proteins such as monoclonal antibodies [23], [24], [25] and streptavidin [26], and that ^99m^Tc can be bound *in vitro* to the complexes using a simple transchelation reaction.

In our current study, we sought to incorporate MT within pIX to determine if a fusion of this type could retain functionality in this context. Our study herein clearly demonstrates our ability to rescue viable adenoviral particles that display functional MT as a component of their capsid surface. The alternative display of MT on the capsid may offer advantages with respect to direct functional applications of this gene product. Our innovative use of a structural fusion protein incorporating MT provides the non-invasive imaging advantages for detecting physical biodistribution and spread of Ad vectors after administration that is not possible employing a reporter gene. Further, the ability to noninvasively observe Ad function on a whole-body level, allows the possibility of detecting virus dissemination outside the tumor site(s) for monitoring clinical safety.

## Materials and Methods

### Cell culture

Human embryonic kidney epithelial (HEK293) cells were obtained from and cultured in the medium recommended by the American Type Culture Collection (Manassas, VA). The HEK293 cell line was grown in high glucose Dulbecco's Modified Eagle Medium (DMEM; Invitrogen; Carlsbad, CA) supplemented with 10% fetal bovine serum (Atlanta Biologicals; Lawrenceville, GA), 100 IU/ml penicillin, 100 µg/ml streptomycin, and 2 mM L-glutamine. The cells were incubated at 37°C and 5% CO_2_ under humidified conditions.

### Animals

Female C57BL6 mice at 4–6 weeks of age were obtained from Charles River Laboratories (Wilmington, MA). All animals received humane care based on guidelines set by the American Veterinary Association. The experimental protocols involving live animals were reviewed and approved by the Institutional Animal Care and Use Committee of LSU Health Sciences Center at Shreveport (Protocol #P-08-017). For the SPECT imaging studies, mice were also pretreated with warfarin (5 mg/kg) dissolved in peanut oil and administered by subcutaneous injection at 3 days and 1 day prior to the Ad vector injections.

### Recombinant Adenovirus Construction

A pIX-MT intermediate sequence in pUC57 was constructed by synthesizing a 932 bp DNA fragment (GenScript; Piscataway, NJ) corresponding to: amino acids 106–140 of the Ad serotype 5 (Ad5) pIX protein coding sequence, followed by a 15 amino acid linker (GGGGSGGGGSGGGGS), amino acids 1–61 of the human metallothionein 1A sequence, a 16 bp DNA linker sequence (TGAGCTAGCGACGTCA), and the 583 bp DNA sequence immediately upstream of the pIX gene (bp 4032–4614; Genbank accession AY339865). This synthetic DNA fragment was subcloned directly into the MfeI - BstXI sites of the AdenoVatorCMV5 shuttle vector (QBiogene; Carlsbad, CA). A turboGFP cDNA sequence (Axxora; San Diego, CA) was also subcloned into the CMV5 expression polylinker site of pAdenoVatorCMV5 for use as a reporter gene. The resulting pAdenoVatorCMV5-tGFP-pIX-MT shuttle vector was used to construct an adenovirus by homologous recombination with pAdEasy1 (containing the E1 and E3 deleted Ad5 backbone) in *E. coli* using methods previously described [27]. The resultant recombinant plasmid was linearized with Pac I and transfected into HEK293 cells to generate the Ad-tGFP-pIX-MT virus. Construction of the Ad-IX-EGFP and Ad-CMV-EGFP vectors used as controls has been described previously [20].

### Virus propagation and purification

Viruses were propagated in HEK293 cells, which do not express wild-type pIX. Viruses were purified by double CsCl ultracentrifugation and dialyzed against Dulbecco's phosphate buffered saline containing 10% glycerol. Final aliquots of virus were analyzed for viral particle (v.p.) titer using absorbance at 260 nm and a conversion factor of 1.1×10^12^ viral particles per absorbance unit (v.p./OD260). Multiplicity of infection (m.o.i.) was determined using an Adeno-X Rapid Titer Kit (Clontech; Mountain View, CA) and represents the number of infectious units (i.f.u.) of virus. Viruses were stored at −80°C until use. The Ad-tGFP-pIX-MT virus titer used was 1.2×10^11^ v.p./mL and 1.4×10^9^ i.f.u./mL.

### Fluorescence Microscopy

Epifluorescence microscopy was performed using an inverted Eclipse TE300 microscope (Nikon Instruments, Melville, NY) equipped with a CoolSNAP fx monochrome CCD camera (Roper Scientific - Princeton Instruments; Trenton, NJ). Images were acquired with a 20X objective and processed with IPLab imaging software (Scanalytics, Inc.; Fairfax, VA).

### Western Blot Analysis

Purified Ad-tGFP-pIX-MT and Ad-CMV-EGFP virions were resolved on precast 4–20% gradient SDS-PAGE gels (Thermo Scientific Pierce; Rockford, IL), and transferred to nitrocellulose membranes. Staining was performed using 1∶500 dilution of an anti-human metallothionein monoclonal antibody (clone UC1MT; Abcam, Inc.; Cambridge, MA) or 1∶500 dilution of a monoclonal anti-adenovirus fiber monoclonal antibody (clone 4D2; Abcam), followed by a 1∶1,000 dilution of a secondary HRP-conjugated goat anti-mouse IgG antibody (Santa Cruz Biotechnology, Inc.; Santa Cruz, CA). Specific protein bands were detected by chemiluminescence using Amersham ECL Plus reagents (GE Healthcare; Piscataway, NJ).

### Assessment of Replication of Virus

Increase in the copy number of the E4 gene was used as a surrogate to determine virus propagation. At 1, 2, 3, and 4 days post-infection of HEK293 cells, aliquots of culture media (300 µL) were removed and frozen at −80°C until use. Total DNA was purified using a QIAamp DNA Mini Kit (Qiagen, Valencia, CA). E4 gene copy number was determined using specific E4 primers and TaqMan probes for real-time polymerase chain reaction (PCR) as previously described [28] with TaqMan fast reagents (Applied Biosystems, Framingham, MA) and measured against a standard curve made from known amounts of adenovirus genome.

### Thermostability assay

Fifty thousand HEK293 cells were subcultured overnight into 24-well tissue culture plates with 1 ml DMEM medium containing 10% FBS. Prior to infection, the viruses were incubated for 0, 15, 45, 60, and 90 min time periods at 45°C, and subsequently, the heat-treated viruses were used to infect the HEK293 cells. At 24 h post infection, the plates were analyzed for relative fluorescence intensity of GFP using a Fluoroskan Ascent Microplate Fluorometer (Thermo Fisher Scientific; Waltham, MA).

### Radiolabeling of virus and gel chromatography

For radiolabeling Ad virions, ^99m^Tc-pertechnetate was reduced in the presence of glucoheptonate using a commercially available kit (DRAXIS Specialty Pharmaceuticals Inc.; Kirkland, Quebec, Canada) for 15 min at room temperature and added directly to aliquots of Ad at a ratio of 10∶1. Incubation with Ads proceeded for 60 min at 37°C. Purification of free ^99m^Tc from labeled adenovirus virions was performed by gel filtration using Sephacryl S-200 HR (GE Healthcare; Piscataway, NJ). Sephacryl resin was loaded into a 20 mL disposable column (Bio-Rad) and pre equilibrated with phosphate buffered saline (PBS) containing 5% glycerol. One mL aliquots of radiolabeled virus were applied to the columns, and 10 drop fractions were collected using an automated fraction collector. The viral particle (v.p.) titer of each fraction was determined by measuring the absorbance at 260 nm in a spectrophotometer, using a conversion factor of 1.1×10^12^ v.p./OD260 [29]. The radioactivity was measured with a dose calibrator (Capintec, Inc.; Ramsey, NJ).

### Metal competition assay

Aliquots of the radiolabeled Ad-tGFP-pIX-MT were incubated in the absence or presence of non-radioactive competitor metal ions. After addition of competitor metals using 100X stock solutions, the aliquots were incubated at room temperature for 15 min. The adenovirus-bound and free ^99m^Tc were separated by gel chromatography using micro spin columns (Bio-Rad; Hercules, CA), and quantified using a NaI (Tl) γ-counter (PerkinElmer Inc.; Waltham, MA).

### HPLC analysis of ^99m^Tc radiolabel stability

Aliquots of the radiolabeled Ad-tGFP-pIX-MT were incubated for 30 min at 27°C in PBS containing 5% glycerol, with the absence presence of 50% mouse serum (Sigma-Aldrich; St. Louis, MO). Aliquot were also incubated for 30 min with 50% mouse serum at increasing temperatures (37, 42, 45, 50, and 55°C). Afterwards, adenovirus particles were analyzed by HPLC as previously described [30] on a Dionex DX500 Chromatography System (Dionex Corp; Sunnyvale, CA) using a strong anion exchange Bio-Monolith QA HPLC column (Agilent technologies; Santa Clara, CA). The HPLC system was equipped with in-line UV (260 nM) and Nal(Tl) gamma scintillation detectors interfaced to a multichannel analyzer. Software analysis was performed for the UV detector using Chromeleon 6.80 (Dionex) and was performed for the gamma scintillation detector using PeakSimple 3.85 (SRI Instruments, Torrance, CA).

### SPECT/CT scanning of Ad-infected mice

Two mice were injected via the tail vein with approximately 1 mCi of the radiolabeled Ad-tGFP-pIX-MT, and image acquisition was started at 60 min afterwards. Similarly, one mouse was injected with approximately 1 mCi of ^99m^Tc-glucoheptonate and one mouse was injected with approximately 1 mCi of ^99m^Tc-pertechnetate. The animals were anesthetized using isoflurane and fixed in a prone position on the bed and center of rotation relative to the gantry of a dedicated small animal trimodality PET/SPECT/CT system (Gamma Medica-Ideas, Northridge, CA). After 60 min, 3D SPECT imaging was performed in a step-and-shoot manner using the following acquisition parameters: 64 projections, 30 seconds/projection (35-minute image acquisition), with a 140 keV photopeak ±10% window. SPECT 3D reconstruction was carried out using Amira software (Gamma Medica-Ideas). Immediately after SPECT imaging was performed, CT images were acquired using the same coordinates as SPECT with 256 projections and 1024×1024 projection matrix size and a voltage of 60 kV; reconstructions were performed using filtered back-projections. Reconstructed data was saved as DICOM files and were imported for further analysis to AMIDE (version 0.9.2; Andy Loening) freeware. Volumes of interest (VOIs) from each image were manually defined and image analyses were performed; the AMIDE ROI statistics tool was used to obtain the mean voxel values for the VOI. The percent activity for each VOI was determined as (VOI mean voxel value×VOI voxel number)/(total image mean voxel value×total image voxel number)×10. 

### Statistical analysis

All data are expressed as means ± SE. *In vitro* experiments were performed in triplicate. Statistical analysis was carried out using Student's t-test, or for multiple comparisons, two-way ANOVA using GraphPad Prism version 5.0 software. Statistical significance was set at P<0.05.

## Results

### Constructing an adenovirus vector containing a pIX-MT fusion

**Figure 1:**
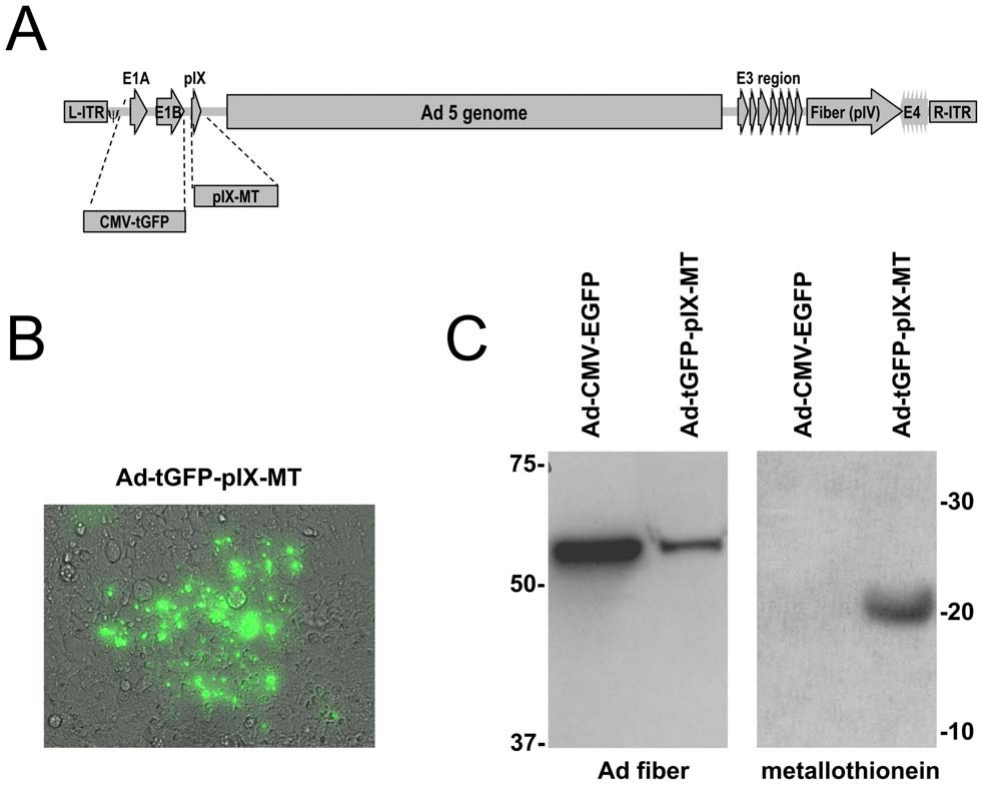
Construction and analysis of a pIX-MT containing adenovirus. (A) Schematic representation of pIX-MT and a CMV-tGFP expression cassette inserted into the Ad5 genome. (B) Expression of GFP in cells infected with Ad-tGFP-pIX-MT shows formation of an individual plaque. (C) Detection of adenovirus fiber and metallothionein proteins incorporated into the purified Ad-tGFP-pIX-MT and Ad-CMV-EGFP viral particles by western blot analysis.

We initiated this work to construct an adenovirus by using an *E. coli* recombination system with a shuttle vector and the adenovirus backbone plasmid. As a labeling system, we incorporated the MT coding sequence as a C-terminus fusion to the pIX protein. We used the *E. coli* recombination system to generate a recombinant replication deficient Ad containing an E1A expression cassette with tGFP sequence under control of the CMV promoter. The resulting recombination inserted shuttle vector sequences containing the expression cassette and pIX-MT fusion gene into the pAdEasy1 adenovirus backbone replacing the E1A and E1B sequences. (*Fig. 1A*). The identity of the recombinant genome was validated by restriction enzyme digestion and PCR analysis and was confirmed by DNA sequencing (data not shown). The resulting pAd5.CMV5-tGFP-pIX-MT plasmid vector was used to rescue an Ad virion by linearization with Pac I and transfection into HEK293 cells. After transfection, positive Ad plaques were detected by fluorescence microscopy (*Fig. 1B*). Propagated Ad-tGFP-pIX-MT virus was purified by double CsCl ultracentrifugation. After virus purification, a recombinant pIX-MT fusion protein was clearly detected as a 21 kD band by western blot analysis of the purified virus using an anti-metallothionein antibody (*Fig. 1C*). This band corresponds to the expected molecular weight of the pIX-MT, and was absent in purified Ad-CMV-EGFP virus. As a control, both the Ad-tGFP-pIX-MT and the Ad-CMV-EGFP virus showed 60 kD bands by western blot analysis using an anti-Ad fiber protein. These results indicate that correct introduction of the MT protein into the viral capsid of an adenovirus by fusing it with the minor capsid protein IX allows the rescue of an infectious virion.

### Analysis of viral DNA replication

To determine DNA replication properties of Ad-tGFP-pIX-MT, the following experiment was performed. Ad E4 copy numbers were analyzed after cells were infected with virus. In brief, 10 i.f.u./cell of Ad-tGFP-pIX-MT, Ad-CMV-EGFP, or Ad-IX-EGFP was used to infect the Human embryonic kidney epithelial (HEK293) cell line. Aliquots of medium were collected on 1, 2, 3, and 4 days post-infection. Total DNA was extracted from the medium (which was used to incubate infected cells) and analyzed for Ad5 viral E4 DNA copy number (*Fig, 2*). Medium from uninfected cells was also obtained at each time point to serve as a base line for viral replication. At 1 day post-infection, the E4 copy number for Ad tGFP-pIX-MT and Ad-IX-EGFP were observed to be approximately 52.6 and 7.5 copies per 100 µL aliquot of medium, while the E4 copy number value for Ad-CMV-EGFP was 12. By 4 days post-infection, Ad5 E4 copy number for each of the adenoviral constructs was dramatically increased to approximately 41,200, 75,900, and 81,700 copies per 100 µL aliquots of media for Ad-tGFP-pIX-MT, Ad-IX-EGFP, and Ad-CMV-EGFP, respectively. Taken together, these data indicate that the adenovirus constructs with modified pIX genes (tGFP-pIX-MT and Ad-IX-EGFP) showed similar replication patterns in 293 cells, and this replication is comparable to an adenovirus construct with a wild-type pIX gene (Ad-CMV-EGFP).

**Figure 2:**
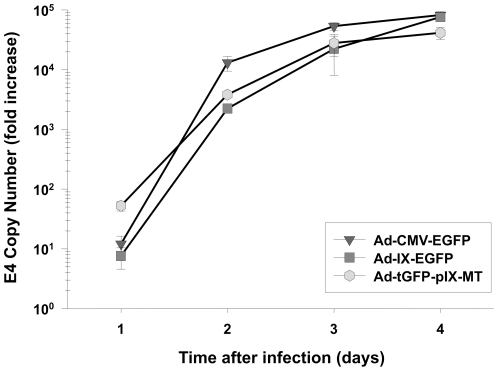
Replication of Ad-tGFP-pIX-MT compared to Ad-CMV-EGFP and Ad-IX-EGFP. Viral copy number was used as an indicator of viral replication in HEK293 cells. Growth medium was collected at 1, 2, 3, and 4 days after infection with Ad-tGFP-pIX-MT, Ad-CMV-EGFP, or Ad-IX-EGFP at an m.o.i. of 10 i.f.u/cell. Each point represents the mean ± SE of three replicate samples.

### Thermostability of Ad-tGFP-pIX-MT virions

**Figure 3:**
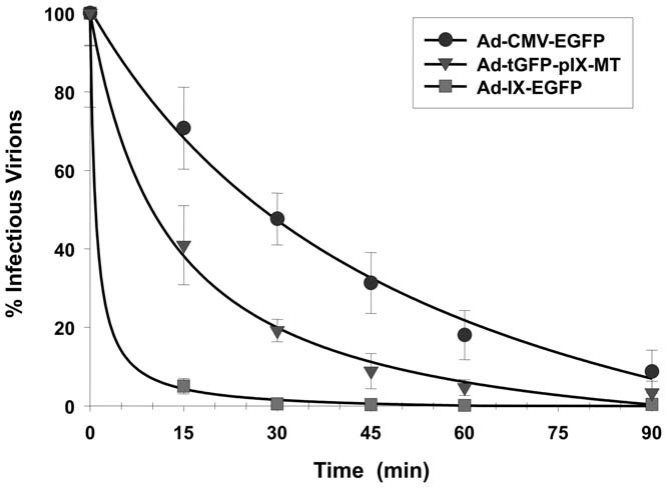
Thermostability of Ad-tGFP-pIX-MT compared to Ad-CMV-EGFP and Ad-IX-EGFP. The thermostability of each virus was determined by incubation at 45°C for various times and quantified in terms of infectious titer. Each point represents the mean ± SE of three replicate samples.

Modification of pIX may destabilize the capsid structure; this possibility was assessed by comparing the thermostability of Ad-tGFP-pIX-MT to a control Ad-CMV-EGFP virus containing the wild-type pIX and to an Ad-IX-EGFP virus incorporating the enhanced GFP (EGFP) protein as a fusion to pIX. Virus samples were incubated at 45°C for various times and then quantified in terms of infectious titer (*Fig. 3*). Beginning at 15 min of incubation, the infectious titer of all viruses decreased in an exponential fashion; the infectious titer for Ad-CMV-EGFP and Ad-tGFP-pIX-MT were 70.8% and 40.4% of initial titers, respectively, while the infectious titer for Ad-IX-EGFP was only 4.8% if the initial titer. Viral titers continued to decrease between 15 and 90 min of incubation, with the time for 50% remaining titer of Ad-CMV-EGFP at 26.7 min, Ad-tGFP-pIX-MT at 11.9 min, and Ad-IX-EGFP at 3.2 min. These results indicate that the MT addition to pIX did negatively affect the thermostability of Ad-tGFP-pIX-MT following exposure of the virus to high temperature compared to a virus containing wild-type pIX. However, when comparing the thermostability curves, this effect was significantly less than that of incorporating a pIX-EGFP fusion (two-way ANOVA, P<0.001).

### Radiolabeling of Ad-tGFP-pIX-MT with ^99m^Tc

**Figure 4:**
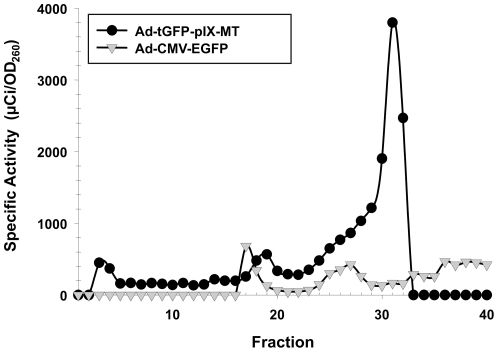
Gel filtration column profile of 99mTc binding to adenovirus containing a pIX-MT fusion protein. After incubation with^ 99m^Tc glucoheptonate, the Ad-tGFP-pIX-MT virus containing a pIX-MT fusion protein and the Ad-CMV-EGFP virus containing a wild-type pIX protein were purified by gravity gel filtration using 20 mL Sephacryl S-200 columns. Each point represents the specific activity in mBq/v.p of individual 10 drop fractions collected.

We tested whether Ad-tGFP-pIX-MT could be radiolabeled by ^99m^Tc binding *in vitro*. In this representative experiment, ^99m^Tc-pertechnetate was reduced in the presence of glucoheptonate for 15 min at room temperature and added directly to the adenovirus solution. Incubation with Ad proceeded for 60 min at 37°C. Purification of free ^99m^Tc from labeled adenovirus virions was performed by gel filtration using a Sephacryl S-200 column. As shown in *Fig. 4*, the specific activity of the peak at fraction 30 corresponding to the highest adenovirus infectious activity was approximately 0.13 mBq/v.p. for the Ad-tGFP-pIX-MT. Importantly, an adenovirus construct Ad-CMV-EGFP containing the wild-type pIX showed lower specific activity at fraction 30 (0.006 mBq/v.p.), possibly from non-specific ^99m^Tc binding to free sulfhydryl groups [31] on the Ad capsid surface. These results demonstrate the feasibility of ^99m^Tc binding *in vitro* to the pIX-MT fusion on the capsid of adenovirus virions using a simple transchelation reaction.

### Metal competition assay

**Figure 5:**
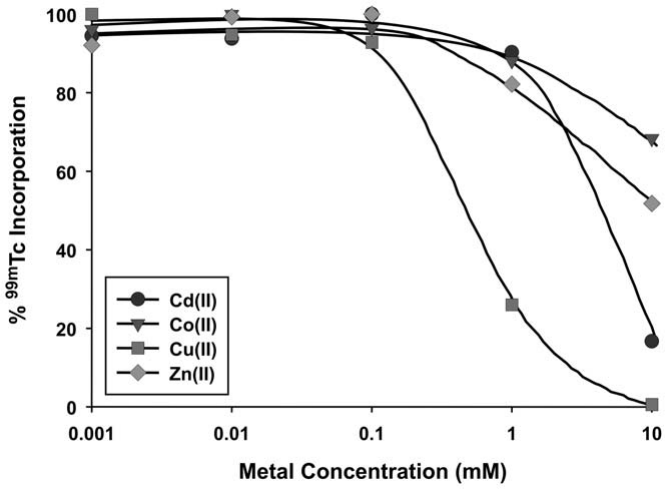
Concentration dependence of competition for metal binding to purified virus. Aliquots of ^99m^Tc-radiolabeled Ad-tGFP-pIX-MT were incubated for 15 min at 37°C with the indicated concentrations of CdCl_2_, CoCl_2_, CuCl_2_, or ZnCl_2_. Virus bound ^99m^Tc was determined counting radioactivity after purification through micro spin columns.

The heavy metals that can be bound by MT include Ag, Au, Bi, Cd, Co, Cu, Fe, Hg, Ni, Pb, Sn, Tc, and Zn, [32], as well as Re [33]. To determine the specificity of ^99m^Tc binding, we performed a metal competition assay using increasing concentrations of CdCl_2_, CoCl_2_, CuCl_2_, or ZnCl_2_. As shown in *Fig. 5*, the bound radioactivity was competed with increasing concentrations of metal, with half maximal concentrations required for displacement of ^99m^Tc. Importantly, these results correlate with the relative order of *in vitro* binding affinities to MT determined to be Cu > Cd > Zn > Co [34]. In addition, Cu was capable of displacing ^99m^Tc from the radiolabeled Ad-tGFP-pIX-MT between 0.1 and 1 mM. However, this concentration of Cu required for displacement is much higher than physiological levels of Cu determined in serum of normal mice, at approximately 400 ng/mL (0.006 mM) [35].

### Stability of ^99m^Tc radiolabel on the Ad-tGFP-pIX-MT virions

**Figure 6:**
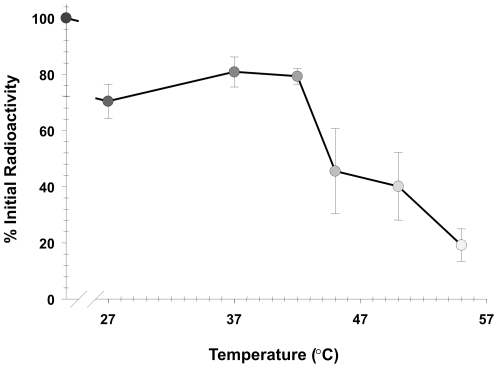
Comparison of ^99m^Tc radiolabel stability on Ad-tGFP-pIX-MT. After 30 min incubation with 50% normal mouse serum at increasing temperatures, samples were analyzed by HPLC on a Bio-Monolith column for ^99m^Tc-radiolabeled Ad-tGFP-pIX-MT in each sample. Radioactivity of peaks corresponding to Ad-tGFP-pIX-MT were quantified and normalized to the radio activity of peaks corresponding to Ad-tGFP-pIX-MT after 30 min in the absence of serum at 27°C. Each point represents the mean ± SE of three replicate samples.

Serum stability of the radiolabel on Ad-tGFP-pIX-MT was investigated by incubating virions for 30 min in 50% normal mouse serum (*Fig. 6*). In the absence of serum, we could detect no loss of radioactivity at room temperature, 27°C or 37°C after incubation for 30 min (data no shown). However, compared with radiolabeled virus incubated in the absence of serum, approximately 80% of the radioactivity was still bound in 50% serum after incubation at 27, 37, and 42°C. This loss of approximately 20% radioactivity at these temperatures may represent loss from low affinity binding sites to ^99m^Tc. When the virus was incubated at higher temperatures, the remaining bound radioactivity was decreased to 46% radioactivity at 45°C, 40% radioactivity at 50°C, and 19% radioactivity at 55°C. This result is similar to the results in *Fig. 3* showing inactivation of virus infectivity at 45°C, as well as previous data demonstrating virus inactivation at temperatures between 48 and 54°C [36].

### SPECT analysis of adenovirus administration *in vivo*

**Figure 7:**
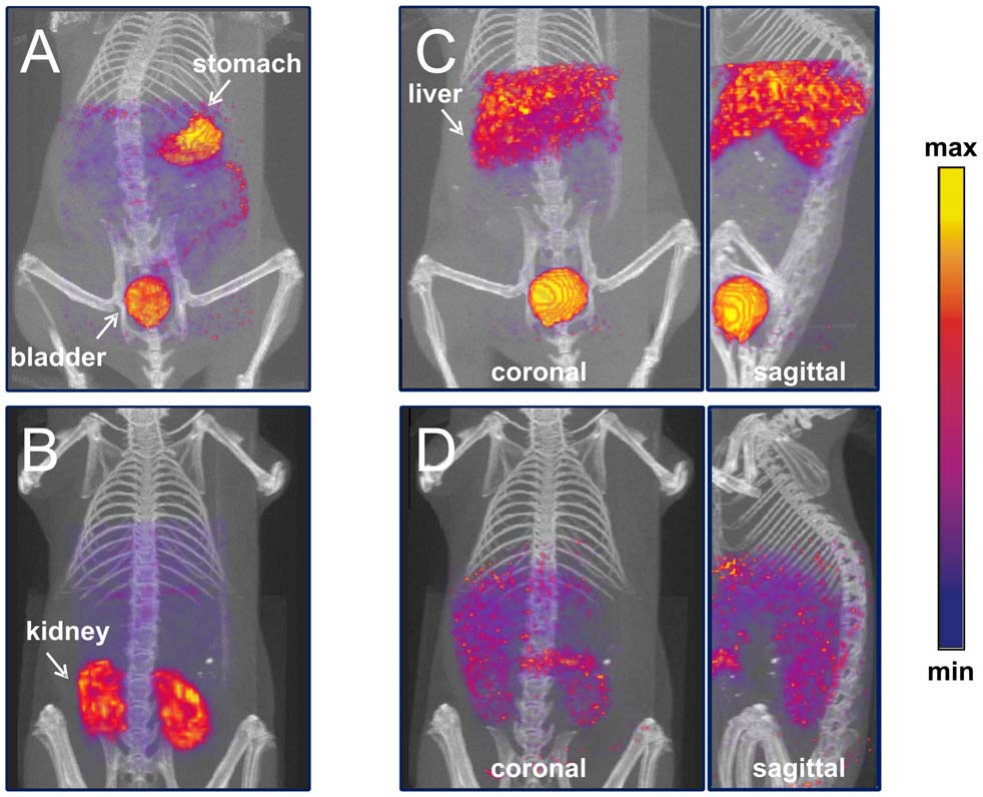
SPECT imaging analysis of a mouse injected with an adenovirus containing a capsid-incorporated pIX-MT fusion protein. C57BL6 mice were injected intravenously with approximately 1 mCi of ^99m^Tc in 0.3 mL PBS, and scanned at 30 min after the injection. Afterwards, 3D renderings of SPECT images in blue-purple-red-yellow scale against CT projections in grey scale were obtained. Shown are: (A) coronal image of a normal mouse injected with ^99m^Tc-pertechnetate; (B) coronal image of a mouse injected with ^99m^Tc-glucoheptonate; (C) coronal and sagittal images of a normal mouse injected with ^99m^Tc-Ad-tGFP-pIX-MT; and (D) coronal and sagittal images of a mouse pretreated with warfarin injected with ^99m^Tc-Ad-tGFP-pIX-MT.

After radiolabeling the Ad-tGFP-pIX-MT, we determined whether SPECT imaging could be used to monitor Ad biodistribution and uptake *in vivo*. In the experiment shown in *Fig. 7*, a female C57BL6 mouse was injected intravenously with ^99m^Tc-labeled Ad-tGFP-pIX-MT. One hour after administration, the animal was imaged; clear localization of activity to the liver (45±8%) was seen (*Fig. 7C*), and bladder activity (32±3%) was also detected. This result strongly supports imaging using pIX-MT, visualizing the normal biodistribution of Ad primarily to the liver upon injection into mice. As a control, *in vivo* imaging of a mouse in *Fig. 7A* injected with ^99m^Tc-pertechnetate alone showed no liver signal, with localization to stomach (14±3%) and bladder (16±2%). Likewise, *in vivo* imaging of a control mouse in *Fig. 7B* injected with ^99m^Tc-glucoheptonate alone also showed no liver activity, with activity localization to kidneys (22±8%).

Liver tropism of administered Ad5 vectors results in rapid sequestration. Key components of this process have recently been elucidated, involving Ad capsid interaction with multiple vitamin K-dependent coagulation factors [37]. Specifically, coagulation factor × (FX) binds to the capsid hexon protein and mediates the majority of the Ad5 liver tropism, presumably via heparin sulfate proteoglycan-mediated pathways [38], [39], [40]. Pretreatment with the drug warfarin, which inhibits vitamin K-dependent coagulation factor production, can deplete circulating FX levels and decrease the liver tropism of i.v. administered Ad5. As shown in *Fig.7D*, warfarin pretreated mice exhibited a decrease in liver activity (14±8%) compared with untreated mice (*Fig. 7C*), as well as a diffuse peritoneal imaging pattern. Together, these data illustrate that the pIX-MT fusion retains its metal binding capacity at the pIX locale and can function in an *in vivo* context, thus making non-invasive imaging analysis feasible.

## Discussion

We have shown herein that our novel pIX-MT labeling strategy for adenovirus yields rescuable virions. The pIX protein is a structural component of the Ad that stabilizes hexon-hexon interactions; there are 240 pIX molecules per virion. With a capacity of 7 metal ions bound per pIX-MT fusion protein, as many as 1680 metal ions can bind per virion. The highly conserved metallothionein structure potentially offers the additional advantage of lower immunogenicity, by “shielding” the Ad from preexisting humoral immunity [41]. Direct western blot analysis of purified Ad-tGFP-pIX-MT produced a protein band that corresponded to a pIX-MT fusion. This data indicates that the pIX-MT fusion was successfully incorporated into the Ad pIX locale. Most importantly, Ad-tGFP-pIX-MT demonstrated specific ^99m^Tc binding *in vitro* when incubated with ^99m^Tc-glucoheptonate. This study also demonstrated our ability to create a virus that encompasses a protein motif useful for SPECT imaging. The majority of previous imaging strategies have focused on incorporating reporter gene using a single imaging modality whereas; our unique virus incorporates the potential for multiple imaging modalities.

Knowledge of biodistribution is crucial in assessing the efficacy of Ad mediated therapies. Clinical trials completed so far have had to rely on conventional histology of biopsy specimens and analysis of body fluids to detect virus; the majority of biodistribution studies have involved PCR-based techniques. For example, after intravenous administration at the highest dose of a replication deficient Ad5CMV-p53, the presence of a unique 89 bp amplicon specific to Ad5CMV-p53 DNA was detectable by Q-PCR in the plasma of 4 of 5 patients at day 14 and 2 of 3 assessable patients at day 28 [42]. In addition, the presence of Ad5CMV-p53 DNA was detected within biopsies of tumor metastasis distant from the intravenous administration site in 6 of 7 assessable patients. However, these results only provide data on the presence of a nucleotide sequence and not on the presence of viral vector particles. In addition, these results highlight the shortcomings of current vector detection methods: the need to acquire multiple biopsies using an invasive procedure that is prone to sampling error and is concomitantly impractical for repeated monitoring of the entire tumor.

Radiotracer imaging technologies that can measure the distribution of radiolabeled tracers *in vivo* are now widely available and have a wide range of research and clinical applications. Two classes of radiotracer imaging systems exist: those designed to imaging SPECT radionuclides such as ^99m^Tc and those designed to image PET radionuclides such as ^64^Cu. Thus, besides SPECT imaging using ^99m^Tc, the pIX-MT could also be used for non-invasive PET imaging, using ^64^Cu binding. SPECT and PET techniques are able to image as low as 10^-10^ to 10^-12^ M of radiolabeled moieties. Each imaging modality has advantages and disadvantages, and thus has specific applications. In general, PET has higher spatial resolution and sensitivity, and is easier to quantify than SPECT. However, SPECT radiotracers are cheaper and much more widely available. Likewise, next-generation SPECT systems have dramatically increased sensitivity. In addition to evaluation of biodistribution by initially *in vitro* ^99m^Tc radiolabeled replication deficient Ads, we expect virions containing the pIX-metallothionein fusion proteins can also be potentially labeled using ^188^Re [33]. This method could offer the advantage of dual-functional SPECT imaging and radionuclide therapy [43], [44], [45].

In total, as hypothesized these data clearly establish the functionality of pIX-MT when incorporated into the adenovirus capsid at the pIX locale. The ability to view real-time molecular biodistribution events in their physiological milieu represents a significant tool to study adenovirus biology *in vivo*. Although *in vitro* studies can serve as a simplified controlled model to study Ad behavior in tumor cells, their simplicity strips them of the complexities of a three-dimensional tumor environment that has profound effects on Ad performance. Ultimately, the highest yield of valuable information about Ads will come from *in vivo* preclinical studies and application of Ad vectors in patients.

It has become clear that cell-specific transductional retargeting of Ad vectors is of fundamental importance with respect to deriving their full benefit in the context of the gene therapies [46], [47], [48]. These transductional retargeting strategies, whereby Ad viruses are designed to selectively infect target cells, have the potential for reducing deleterious side effects and increasing the therapeutic index of these agents. The pIX-MT labeling system demonstrated herein, offers the potential for noninvasive dynamic imaging of Ad biodistribution that can be easily used by existing nuclear medicine imaging modalities. Importantly, this tool can be used for preclinical development of transductional retargeted Ads, and it would be practical in the clinical setting to monitor the therapeutic application of these agents.
